# Supplementary material for: Brown and Beige Adipose Tissue: One or Different Targets for Treatment of Obesity and Obesity-Related Metabolic Disorders?
Source: Int J Mol Sci. 2024 Dec 11;25(24):13295. doi: 10.3390/ijms252413295 (PMC11677471; doi:10.3390/ijms252413295)
Supplement: Supplementary file 1 [file ijms-25-13295-s001.zip › TableS1.pdf]

	Impact in People on:				The Main Mechanism of BAT Activation	Influence on		SE, Mechanism	References
**Class/Drug**	**BM**	**lipids**	**Glucose and IR**	**BP**		**Beiging /rBAT**	**Browning / cBAT**		
Triiodothyronine (T3)	↓	↓ TH, LDL-C, TG	↔↑ Fasting glucose, IR	↑sBP	Binding to TGR receptors activates it, leading to the formation of a heterodimeric complex with the retinoic acid receptor and binding to the transcriptional coactivator *PPAR-γ PGC-1α*. TRC also interacts with the cyclic response element-binding protein (CREB), acting synergistically with norepinephrine and increasing the expression of *UCP1*.	↑	↑↑	Activation of the TRH receptor in other tissues (myocardium, vascular wall) ↑ gene expression involved in cardiac contraction and smooth muscle contraction , as well as arrhythmogenesis and others. ↑ BP, heart rate, HLV, AF, PAR in liver: ↑ gluconeogenesis, ↑ glucose levels in fasting conditions and ↑ insulin resistance.	(29-46)
β3AR agonists, CA	↓	↓ TG, ↑ HDL	↓↑ glucose, IR	↑ sBP	Activation of β3-adrenergic receptors (ADRB3) via the p38-MAPK signaling pathway, followed by binding	↑	↑↑	Activation of ADRB3 in other tissues (myocardium,	(48,49,50-63)

					of cAMP to ADRB3 in brown adipocytes, results in increased intracellular cAMP and subsequent activation of cAMP-dependent protein kinase A. This enzyme then phosphorylates target proteins and genes responsible for uncoupling mitochondrial respiration, as well as mediating lipid oxidation and the release of free fatty acids, which activate *UCP1* through ↑ *PGC1α*.			vascular wall)→ ↑ gene expression involved in cardiac contraction and smooth muscle contraction, аритмогенез as well as arкhythmogenesis and others.<br>↑ BP, heart rate, HLV, AF, PAR.<br>in liver: ↑ gluconeogenesis, ↑ glucose levels in fasting conditions and ↑ insulin resistance.	
PPAR agonists Rosiglitazone, pioglitazone	↑	↓ TG, ↑ HDL	↓ glucose, IR	↔	Browning is induced through the signaling pathways MAPK and PI3, the formation and activation of the *PPARγ/PRDM16/EBF2/EHMT1* complex ensures differentiation of precursors into beige adipocytes. Beiging is induced by activating the SIRT1-PRDM16 pathway.	↑, in CR pioglitazone -↓	↑↑	↑ weights, ↑ fluid retention, ↑ bone resorption through the activity of *PPARy* in other tissues	(83,84,88,90,91)
PPAR-α	↔	↓	↔	↔	Activation of *PPARα* plays a	↑↑	↑	Muscle pain and	(92-96)

agonists (fenofibrate)		TH, LDL -C, ↓↓ TG, ↑ HDL	glucose , ↔/↓ IR		dominant role in regulating the transcriptional activity of *UCP1.* ↑ expression of the coactivator *PPARγ PGC1α* and irisin, with subsequent ↑ *PRDM16, ADRB3*, *BMP8B* and *UCP1* in brown adipocytes. ↑ production of FGF21 → browning.			spasms, nausea, vomiting, abdominal pain	
Selective modulator PPAR-γ (glivec)	↔			↔	↑ expression of thermogenic genes (*UCP1, PGC1a, COX-5b*) and expression of beige adipocyte markers (*CD137 and TMEM26)*	↑↑	↑	Leukopenia, anemia, thrombocytopeni a, nausea, vomiting, fluid retention, diarrhea, bleeding, fever, muscle cramps and bone pain.	(103)
Metformin	↔↓	TH, ↓ TG,	↓ glucose , insulin and IR,	↔↓ BP	Activates *AMPK,* SIRT1 and 3 (AMPK/SIRT1/PGC1α pathway); through their activation, it induces beiging. ↑ brown fat mass, ↑ expression of thermogenic markers in it and ↑ adipogenesis and thermogenesis through the αlAMPK-PRDM16 signaling pathway; activates α1AMPK in the intestine and provides communication within internal brown fat tissue. Involves mTOR and FGF21	↑↑	↑	Nausea, abdominal pain, dyspepsia.	(3,24,68-82)

					production.				
Resveratrol	↓↔ (dose-dependent)	↓ TH, TG	↓ glucose, ↓ IR	↔ BP	Promotes the formation of beige adipocytes by activating SIRT1 through direct deacetylation of *PPARγ* recruiting *PRDM16* (AMPK/SIRT1/PGC-1α pathway and SIRT1-PPAR-γ), both by maturing preadipocytes and inducing the process of transdifferentiation of mature white adipocytes.	↑↑	↑	no	(65-66)
arGPP1 exenatide, liraglutide, semaglutide	↓*	↓ TH and TG	↑ insulin, ↓ glucose	↓ BP,	Induces thermogenesis in BAT and rBAT; activates AMPK with involvement of NO in the ventromedial hypothalamic nucleus; ↑ sympathetic innervation of GT.	↑	↑	Nausea, vomiting, gastrointestinal motility disorder, pancreatitis	(88,89,107-112,114)

↑ increase/enhancement; ↓ decrease/reduction; AF - atrial fibrillation; AMP - 3',5'-cyclic adenosine monophosphate; AMPK - AMP-activated protein kinase; BAT - brown adipose tissue; *BMP8B* - Bone morphogenetic protein 8B; BP - blood pressure; CA – catecholamines; cBAT - classical brown adipose tissue; *CD137* - Cluster of differentiation 137; *COX-5b* - Cyclooxygenase-5b; CR - clinical research; CREB - cyclic response element-binding protein; EBF2/EHMT1 - EBF2 (early B-cell factor 2) protein and EHMT1 (euchromatic histone-lysine N-methyltransferase 1); FGF21 - Fibroblast growth factor 21; GT – gastrointestinal tract; HDL - high-density lipoproteins; HLV- hypertrophy of the left ventricle; IR - insulin resistance; LDL-C - low-density lipoproteins; NO – nitric oxide; PAR–pulmonary artery pressure; *PGC-1α—PPARγ* coactivator 1α; *PPAR-γ* peroxisome proliferator-activated receptor gamma; *PPAR*—proliferator-activated receptor; *PRDM16*—PR domain containing 16 transcription factor; rBAT - recruitable brown adipose tissue; SE - side effects; SIRT1—sirtuin 1; SIRT3—sirtuin 3; TG – triglycerides; TH - total cholesterol; TGR - Thyrotropin-releasing hormone receptor; TH - total cholesterol; *TMEM26* - Transmembrane protein 26; TRC (TRP-1/2 channel-related protein) is a protein that forms a complex with the retinoic acid receptor and *PPAR-γ PGC-1α;* TRH(thyrotropin-releasing hormone) TRH receptor is a G-protein-coupled receptor that binds to TRH; *UCP1* - uncoupling protein 1
