# Supplementary figures and images for: Brown and Beige Adipose Tissue: One or Different Targets for Treatment of Obesity and Obesity-Related Metabolic Disorders?
Source: Int J Mol Sci. 2024 Dec 11;25(24):13295. doi: 10.3390/ijms252413295 (PMC11677471; doi:10.3390/ijms252413295)

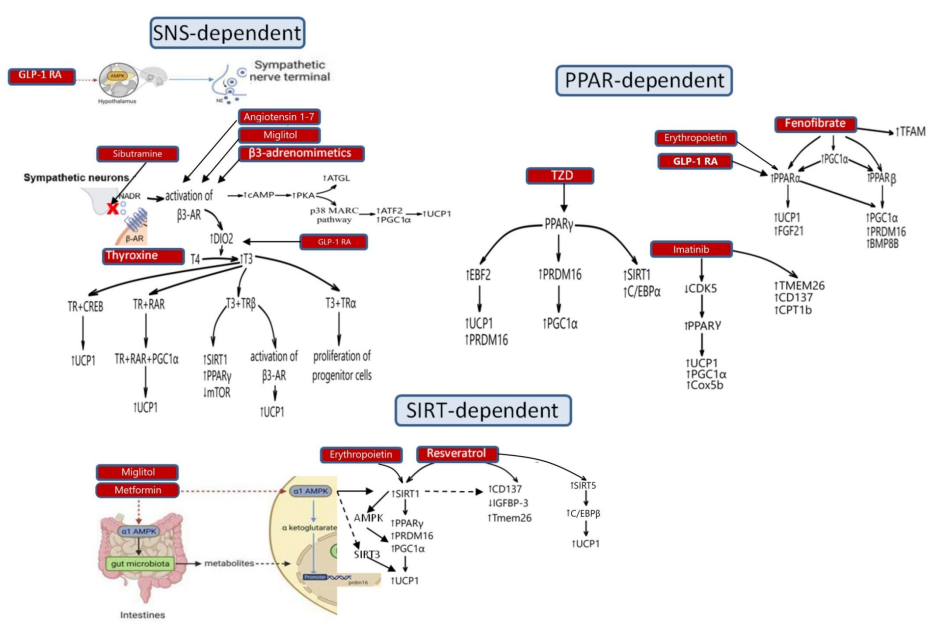

Supplement: Supplementary file 1 [file ijms-25-13295-s001.zip › Figure S1.png]
